# Shared Decision-Making in Patients With Prostate Cancer in Japan: Patient Preferences Versus Physician Perceptions

**DOI:** 10.1200/JGO.2016.008045

**Published:** 2017-04-13

**Authors:** Ulrike Schaede, Jörg Mahlich, Masahiko Nakayama, Hisanori Kobayashi, Yuriko Takahashi, Katsuhiko Saito, Hiroji Uemura, Masayuki Tokumitsu, Kazutake Yoshizawa

**Affiliations:** **Ulrike Schaede**, University of California at San Diego, San Diego, CA; **Jörg Mahlich**, Janssen Pharmaceutical K.K., Tokyo, Japan, and University of Düsseldorf, Düsseldorf, Germany; **Masahiko Nakayama**, **Hisanori Kobayashi**, and **Kazutake Yoshizawa**, Janssen Pharmaceutical K.K.; **Yuriko Takahashi** and **Katsuhiko Saito**, Anterio, Tokyo; **Hiroji Uemura**, Yokohama City University, Medical Center, Yokohama; and **Masayuki Tokumitsu**, Kitasaito Hospital, Ashikawa, Japan.

## Abstract

This article adds the Japanese perspective to our knowledge of shared decision-making (SDM) preferences by surveying patients with prostate cancer (PCA) and physicians in Japan. In 2015, 103 Japanese patients with PCA were asked about their SDM preferences by using an Internet-based 5-point-scale questionnaire. Concurrently, 127 Japanese physicians were surveyed regarding their perceptions of patient preferences on SDM. Drivers of preferences and perceptions were analyzed using univariable ordinal logistic regression and graphing the fitted response probabilities. Although 41% of both patients and physicians expressed and expected a desire for active involvement in treatment decisions (a higher rate than in a similar study for the United States in 2001), almost half the Japanese patients preferred SDM, but only 33% of physicians assumed this was their choice. That is, 29% of Japanese physicians underestimated patients’ preference for involvement in making treatment decisions. Patients with lower health-related quality of life (as measured by the Functional Assessment of Cancer Therapy-Prostate [FACT-P]) expressed a stronger preference for SDM. The study shows that the worse the medical situation, the more patients with PCA prefer to be involved in the treatment decision, yet physicians tend to underestimate the preferences of their patients. Perhaps in contrast to common assumptions, Japanese patients are as interested in being involved in decision making as are patients in the United States.

## INTRODUCTION

“Just as they need air to breath, humans need to have influence.” This management wisdom is often applied in well-run workplaces around the world and also applies when humans become patients. Several studies conducted in the United States dating back to the 1980s and 1990s showed that patients felt a need to be involved in making treatment decisions and that shared decision making (SDM) improves compliance with treatment regimens and the overall patient situation.^1-7^

In spite of these insights, a seminal 2001 study by Bruera et al^8^ on patients’ preferences versus physicians’ perceptions of those preferences showed marked differences in the degree to which both sides saw the desired amount of involvement. In that palliative care study, physicians were divided into three roughly equal groups according to whether they thought patients wanted to be active, passive, or share in decision making. However, 63% of patients preferred to participate in decision making, 20% wanted to be active, and only 17% wanted to be passive. The authors concluded that each patient should be assessed proactively for individual preferences.

Our study in Japan replicates the Bruera et al^8^ survey with two major differences. First, we focused only on patients with prostate cancer (PCA) at various stages of disease progression. This allowed for more specific identification of patients, because PCA treatment options involve significant trade-offs and lifestyle decisions. Second, our survey was not of matched physician-patient pairs. Rather, it was of patients and physicians who were not associated, and it was countrywide and across different types of hospitals. This generalized the study and removed any possible response bias in small physician-patient pairs. Still, the similarities in survey design allowed for a comparison of patients’ and physicians’ attitudes in the United States and Japan.

Japan is an interesting case for three reasons. First, the stereotypical albeit dated image of health care in Japan is that physicians (referred to with the honorific “sensei” or teacher) dictate medical decisions and often do not discuss treatment options with their patients.^9^ Cultural reasons are often invoked to describe a rather passive attitude of patients in health care counseling.^10,11^ We are interested in whether this stereotype (and possible assumptions about Asian patients in general) and the practice itself truly reflect the interests of Japanese patients today.

Second, Japanese society has changed considerably in terms of the wide dissemination and adoption of the Internet and an increase in information, individualization of preferences, and conceptualization of personal rights.^12^ In reaction to these changes, Japanese medical schools have begun to include classes in communication skills in their curricula.^13-15^ On the patient side, a 2012 laboratory study of the preferences of college students regarding treatment of non–life-threatening infections suggested no marked differences between US and Japanese students.^16^ Our study aimed to capture ongoing changes in Japan and extend existing research to patients with PCA.

Third, patients with cancer in Japan are mostly treated by specialty physicians rather than oncologists (eg, urologists in the case of PCA). Although a professional oncologist certification was introduced in 2006, based on training and at least 5 years of experience in clinical oncology,^17^ the specialty orientation persists, and the main differentiation drawn in Japan is between specialty physicians at general hospitals compared with specialty physicians at specialized cancer hospitals. The latter are generally considered to be more open to patient counseling and involvement. We explored differences in SDM assessments between hospital types.

## PATIENTS AND METHODS

We developed an online survey for patients and physicians that replicates the questionnaire designed by Bruera et al.^8^ Survey participants were selected by Intage, a Tokyo-based medical market research firm.

### Survey Sample

An invitation to participate was sent by e-mail to patients with PCA who were registered in the Intage data bank. Participants were selected on the basis of diagnosis, pharmacotherapy, and frequency of hospital visits. These filters resulted in a sample of 2,622 men being invited to participate in the survey between August and November 2015; 103 men responded. Although an Internet-based survey may translate into self-selection by those who are more interested in access to information, the age distribution of patients in our sample does not suggest a bias toward younger men (Table 1).

**Table 1:**
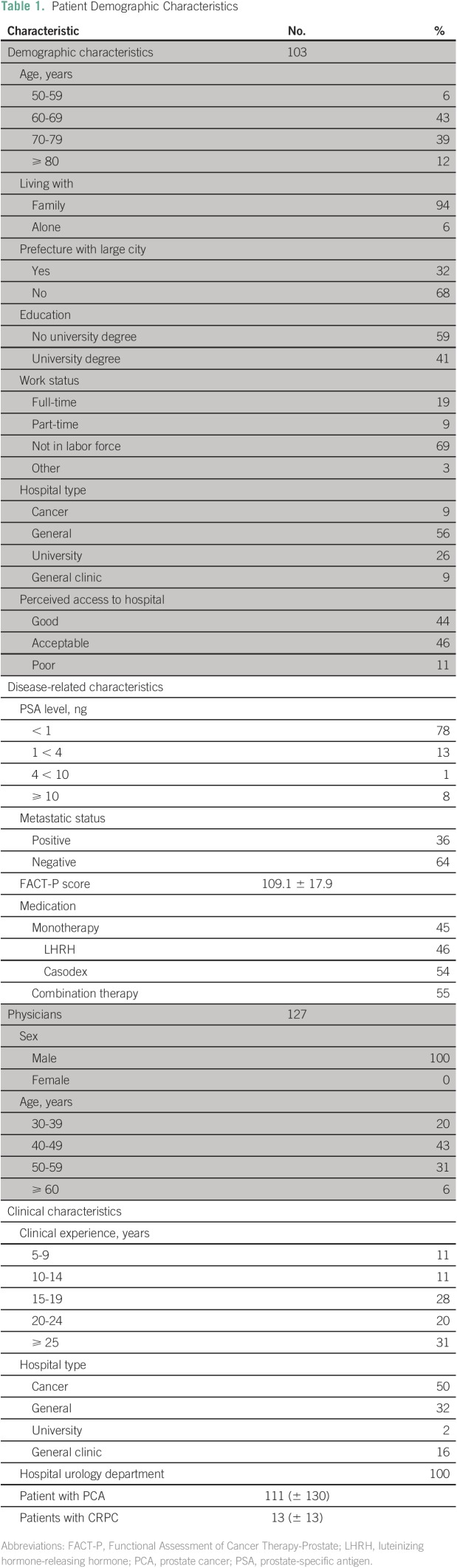
Patient Demographic Characteristics

Concurrently, an invitation to participate was sent by e-mail to 44,400 physicians in Japan. Inclusion criteria were specified in the e-mail: participants needed to work in a urology department, with a minimum of 5 years of clinical experience. In addition, the survey was limited to physicians who served at least 10 patients with PCA and allocated at least 50% of their time to medical consultation. Among those who fulfilled these criteria, 127 physicians answered the questionnaire, and of these, three declined to answer questions on preferences.

### Questionnaire

The survey consisted of three sections. The first section contained questions on the patient’s and the physician’s background (Table 1).

For the second section regarding SDM, given our interest in a comparison between the United States and Japan, we used the same survey questions as Bruera et al.^8^ In their questionnaire, patients were asked to select one from the following statements:

1. I prefer to make the treatment decision on my own.
2. I prefer to make the treatment decision after hearing the physician’s opinion.
3. I prefer to make the treatment decision together with the physician.
4. I prefer the physician to make the treatment decision after talking to me.
5. I prefer the physician to make the decision on his/her own.
6. I don’t know.
7. I prefer not to answer.

To assess the health-related quality of life of patients in our survey, we also included a set of questions based on the Functional Assessment of Cancer Therapy-Prostate (FACT-P), which can be accessed at the Web site of the Functional Assessment of Chronic Illness Therapy (FACIT) Measurement System. The FACIT Measurement System is a collection of quality-of-life questions targeted to the management of chronic illness that covers physical, social, emotional, and functional well-being. We used the score from the Japanese version of the FACT-P survey to control for differences in progression of the disease.

Third, the physicians in our survey were asked to select one from the following statements:

1. I think the patient prefers to make the treatment decision on his own.
2. I think the patient prefers to make the treatment decision after hearing the physician’s opinion or input.
3. I think the patient prefers to make the treatment decision together with the physician.
4. I think the patient prefers that the physician make the treatment decision after talking with the patient and hearing the patient’s opinion.
5. I think the patient prefers the physician to make the decision on his own.
6. I don’t know.
7. I prefer not to answer.

Since the 2001 Bruera et al^8^ study, the SDM research field has reduced this answer set to three main categories: (1) “active” (answers to questions 1 and 2); (2) “shared” (answer to question 3); and (c) “passive” (answers to questions 4 and 5).

The 2001 survey questions regarding SDM were translated into Japanese independently by two native Japanese speakers. Quality and essence capture were then validated by reconciling the two translations into one questionnaire that was translated back into English by two native English speakers, and their version was again translated into Japanese. A group of 35 patients and five physicians confirmed the appropriateness of our translation. The survey is in Japanese and is available upon request.

### Statistical Analysis

Questionnaire responses were summarized into frequencies and ratios. The significance of differences between patient and physician preferences was determined through χ^2^ analysis. We explored the association between patient and physician characteristics and decision-making preference using univariable ordinal logistic regression and maximum likelihood.^18^ Note that ordinal logistic regression assumes proportional odds (ie, the coefficients describing the relationship between the lowest *v* all higher categories of the response variable are equal to the coefficients that describe the relationship between the next lowest category and all higher categories). The coefficients are to be interpreted as the log odds of preferring a more active decision-making style. A two-sided *P* value < .10 was considered significant. All statistical analyses were conducted with JMP 9.0 (SAS Institute Japan, Tokyo, Japan).

## RESULTS

Table 1 summarizes the characteristics of the sample population. Of the patients, 49% of respondents were younger than age 70 years, and almost all (94%) lived with family members. Sixty-eight percent lived in prefectures without a large city (defined as a city with a population of more than 1 million), indicating a more rural and traditional lifestyle. Only 9% were treated in a specialized cancer hospital. Of the total, 72% were not in the labor force. Patients’ prostate-specific antigen levels were, on average, less than 1 ng/mL. The average score for the FACT-P was 109. The sample was roughly evenly divided between patients receiving single-drug treatments and patients receiving combination-drug treatments typically prescribed for advanced-stage cancers.

Of the physicians, 63% were younger than age 50 years, and the median length of clinical experience was 15 to 19 years. All worked in specialty urology departments, half in general hospitals (including university hospitals and general clinics); the other half in specialized cancer hospitals. On average, they treated 111 patients with PCA and 13 patients with castration-resistant PC. All physicians were male, reflecting the study focus on PCA, as well as Japan’s system whereby patients with cancer are primarily treated by specialty physicians rather than oncologists; as in the United States, urology is a specialty dominated by male physicians.

**Fig 1:**
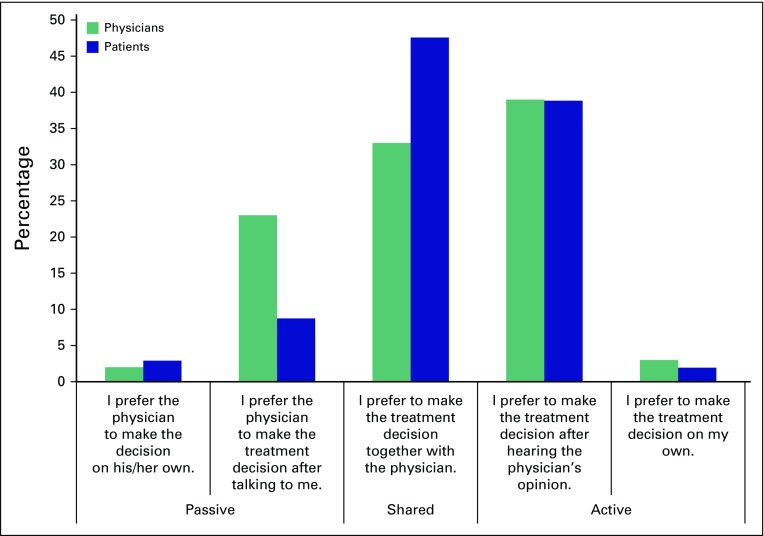
Preferences of patients (n = 103) and physicians (n = 124).

Questionnaire responses are reported in Figure 1, similar in form to Bruera et al.^8^ Patients’ responses show a distribution skewed heavily toward active SDM: 89% expressed strong interest in SDM, with 41% wanting to be actively involved in the decision, and 48% preferring SDM. Only 11% of patients wanted the physician to determine the treatment course.

In contrast, the physicians’ responses were distributed more evenly across the spectrum: 41% of physicians assumed that patients preferred to be actively involved (a direct match), and 33% assumed that patients wanted to share the process. Meanwhile, 26% of physicians thought that patients wanted them to make the decision based upon their joint discussions, and 3% thought the patients wanted them to decide unilaterally without consultation. This means that a quarter of physicians overestimated the patients’ preference for passivity. The differences between patient preferences and physician assumptions are significant (χ^2^ test *P* = .0158).

To identify factors driving these preferences and perceptions, we conducted a univariable ordinal logistic regression analysis, with the decision-making preference as the dependent variable. Active preferences have a higher value than passive ones. Table 2 reports the coefficients of the ordinal logistic regression, using a maximum likelihood estimator.

**Table 2:**
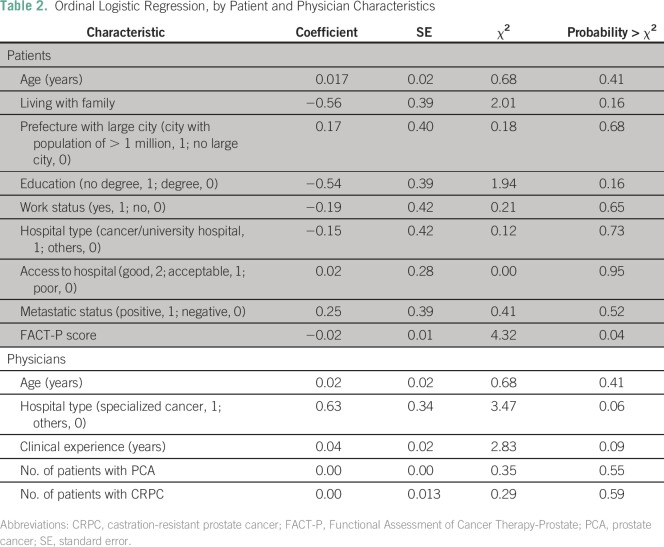
Ordinal Logistic Regression, by Patient and Physician Characteristics

**Fig 2:**
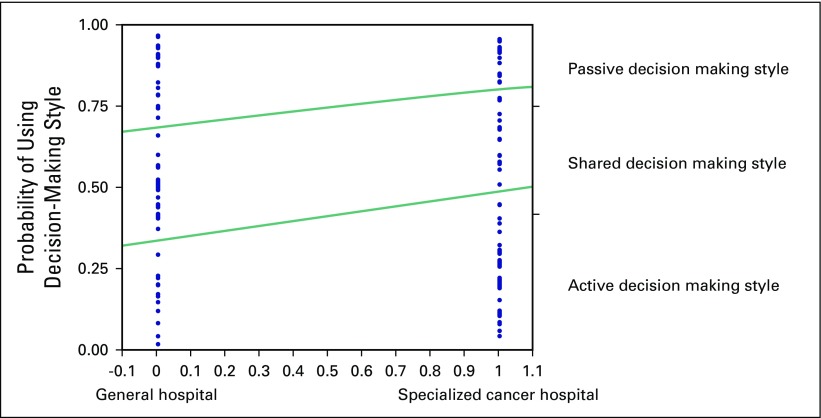
Cumulative logistic probability plot for differences between specialized cancer hospitals and other hospitals.

The lower portion of Table 2 shows that more experienced physicians were more likely to assume that their patients preferred active involvement (effect likelihood ratio test *P* = .09). Moreover, the difference between specialized cancer hospitals and general hospitals (general and university hospitals and general clinics) was associated with a 0.63 increase in the log odds of assuming that patients had a preference for more active decision making. To explore this further, Figure 2 plots the regression results for differences between general hospitals and cancer hospitals. The distance between the lines represents the fitted response probability for the levels, in the order of levels indicated on the right-hand y-axis. Physicians at specialized cancer hospitals were more likely to assume that patients wanted to be actively involved in the medical decision making compared with their colleagues at general hospitals and in particular small private practice clinics (*P* = .06).

**Fig 3:**
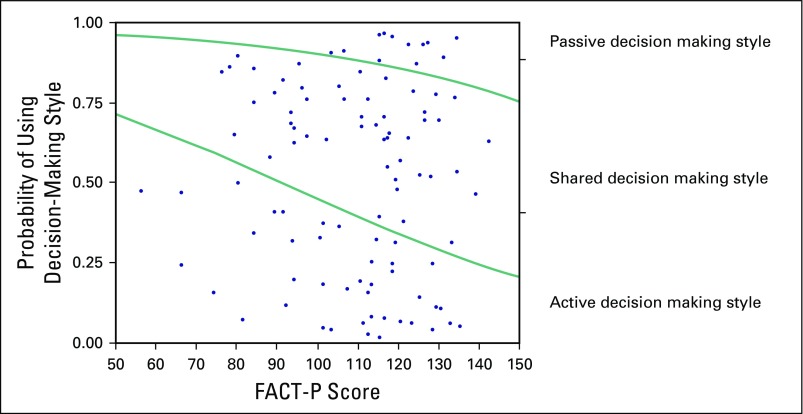
Cumulative logistic probability plot for the association between FACT-P and SDM preferences.

Returning to Table 2, most results for patients are insignificant, with one important exception: there was a negative association between FACT-P and active decision preferences at *P* = .04. Figure 3 shows fitted response probabilities that illustrate the relation between FACT-P and SDM preferences. Patients with a higher FACT-P score (ie, those who feel better) were more likely to prefer a passive decision-making approach.

Table 3 reports results of further analysis of this negative association by showing the differences in means for each of the three preference categories. Recall that the mean FACT-P score for the total sample was 109. Moreover, Cella et al^19^ showed that the minimal clinical difference in FACT-P is between 6 and 10. In our study, the difference between passive preference (mean score of 119.7) and shared preference (109.5) exceeds the minimal clinical difference. The difference in mean scores between passive and active (119.7 *v* 105.6) is also noteworthy at the clinical level.

**Table 3:**
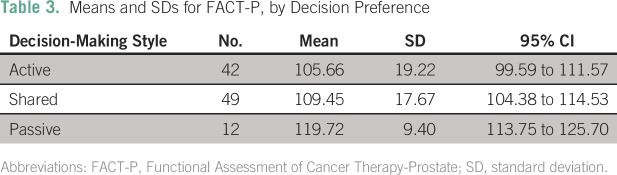
Means and SDs for FACT-P, by Decision Preference

## DISCUSSION

Overall, our results for Japan are similar to those of previous studies for the United States,^8,20^ which found that approximately 83% of patients with cancer in North America preferred to have active or at least SDM, and 68% of physicians assumed that that was the case. This compares with 89% and 75%, respectively, in our study in Japan. It is possible that our focus on PCA affects the results, because it has been shown that patients with PCA express a preference for more active participation compared with patients with other cancers.^21^ However, whereas the Bruera et al^8^ study included only palliative care patients, our study surveyed patients with early- as well as advanced-stage disease, and we found that the latter preferred more involvement. If anything, the inclusion of patients with early-stage disease could have shifted the distribution toward less preference for SDM, but this was not the case.

In light of the persistent image of Japanese physicians as unilateral decision-makers and patients as order-takers, our findings may be considered surprising. As suggested by other Japanese researchers,^11^ Japanese patients have become interested in being included in the treatment decision-making process, and Japanese physicians have become attuned to patients’ preferences.

That said, in our study, 29% of physicians underestimated patients’ desire to be involved (compared with 30% in the US study of 2001). Regarding factors that determined physicians’ perceptions in Japan, we found that clinical experience significantly shaped assumptions: the more experienced a physician was, the more likely he was to assume that patients preferred active involvement. Moreover, physicians in specialized cancer hospitals were more likely to assume that patients had a preference for active decision making. This matches the reputation of Japanese specialized cancer hospitals for offering more interaction and consultation with patients. This finding reflects the particular setup of cancer care in Japan, and stands in contrast to a recent US study showing that general practitioners assumed that their patients had higher SDM preferences than practitioners in specialized hospitals.^22^

Turning to patient preferences, we find that patients with a higher FACT-P score (meaning higher reported quality of life) were more inclined to delegate medical decisions to the physician. This suggests a relationship between the criticality of the situation and patient preferences: the worse the patient feels in terms of physical, social, emotional, or functional well-being, the more the physician should assume that the patient has a preference for involvement in medical decision making. This result is the direct opposite of the results of existing studies. In Japan, for example, it has been reported that patients with life-threatening diseases prefer to delegate medical decisions to the physician.^23^ For women in the United States, research shows that better self-rated health is associated with a preference for more active decision making.^24^ Yet the results for our sample are strong, at the clinical relevance level.

Moreover, we report mild numerical support (although it is not statistically significant) that being in the labor force is associated with preference for a passive role in decision making. Perhaps working patients are not as interested in investing in the decision-making process. We also find a weak association between lower education level and a preference for a passive role in decision making. This echoes research in Australia and the United States, suggesting that people in lower-skilled occupations are significantly associated with a preference to delegate medical decision making.^25-27^

With regard to age, our results do not support results from western countries that younger patients prefer more active roles.^3^ This may be surprising, given assumptions about Japanese patients and physicians, because a traditional passive attitude should be more pronounced for older patients. The lack of a strong finding for Japan may be a result of the fact that our study was small and focused on male patients with PCA, where treatment options mean making significant lifestyle choices.

Although our focus on PCA allowed for detailed insight into SDM choice depending on stage of treatment, our results may differ from those for other cancer types, especially with regard to life expectancy. Future research may reveal differences across physician specialties^28^ in Japan.

Finally, it has been suggested that female physicians tend to be more attuned to patient needs and report higher scores in communication skills than male physicians^29^ and that SDM plays an important role in the treatment of female patients.^30-32^ Given our focus on PCA, and therefore the sample bias on only male physicians and patients, we cannot speak to the effects of sex on SDM matching in Japan. In recent years, the sex distribution of physicians in Japan has begun to approach US levels, with approximately 30% of physicians being female. We leave it to future research to explore SDM matching in Japan by the sex of patients and physicians.

Overall, our study offers two main insights for treatment of PCA. The worse the patient’s well-being and medical situation, as assessed through tools such as FACT-P, the more patients with PCA prefer to be involved in treatment decision making, even though physicians tend to underestimate their patients’ desire for participation. Moreover, Japanese patients, although they may appear to be polite or even submissive, are as interested in shared or active decision making as are Americans.
